# Bioformulations of *Entrophospora lutea* enriched with biostimulants for growth promotion and control of *Rhizoctonia* root rot in lupine

**DOI:** 10.1038/s41598-026-66529-7

**Published:** 2026-08-19

**Authors:** Marwa A. M. Atwa, Sozan E. El-Abeid

**Affiliations:** 1 Legume and Forage Diseases Research Department, Plant Pathology Research Institute, Agricultural Research Center, ARC, Giza, Egypt; 2 Mycology and Plant Diseases Survey Department, Plant Pathology Research Institute, Agricultural Research Center, ARC, Giza, Egypt

**Keywords:** *Entrophospora lutea*, Lupine, Biostimulants, Formulation, *Rhizoctonia solani*, Biotechnology, Microbiology, Plant sciences

## Abstract

Arbuscular mycorrhizal fungus (AMF) *Entrophospora lutea* was previously selected as a highly compatible isolate for lupine, and the main objective of the present study was to further improve its performance and practical applicability by developing two *E. lutea* formulations using peat and vermiculite carriers enriched with various biostimulants. Incorporating biostimulants into AMF formulations can enhance fungal performance and root symbiosis. The first formulation contained proline, ascorbic acid, and hemicellulose, whereas the second contained proline, humic acid, and mannitol. Both formulations improved AMF performance by increasing the number of infective propagules and root colonization rate. Their efficacy against *Rhizoctonia* root rot in lupine was evaluated in comparison with unamended *E. lutea* and the fungicide Rizolex under greenhouse and field conditions. Under greenhouse conditions, Formulation 2 increased plant survival to 92% and reduced disease severity to 24.2%, compared with 48% survival and 65.8% disease severity in the infected control. Root colonization reached 78% and 69% for Formulations 1 and 2, respectively, compared with 53% for the unamended *E. lutea*. These improvements were associated with enhanced plant growth, nodulation, nitrogenous activity, and enhanced antioxidant enzyme activities. Formulation 2 showed a more pronounced effect with increased proline accumulation and caused 82.6% reduction in H_2_O_2_ accumulation compared with the infected control. Under field conditions, both formulations improved yield compared with the untreated control across the two growing seasons. No significant differences were observed between the AMF formulations and Rizolex for most evaluated parameters. These findings demonstrate that the developed biostimulants-enriched AMF formulations are effective and environmentally friendly alternatives to chemical fungicides for controlling *R. solani* root rot in lupine, while improving plant growth and productivity.

## Introduction

Arbuscular mycorrhizal fungi (AMF) are a ubiquitous group of soil microorganisms that live in symbiosis with most plants, playing an important role in sustainable agriculture as natural biostimulants^1^. AMF promote plant growth by enhancing the uptake of water and nutrients from the soil^2^. Their extensive mycelial network extends beneath the roots, effectively increasing the root surface area and helping plants access a larger volume of soil^3^. Additionally, AMF are regarded as an economical and effective group of plant growth-promoting microorganisms (PGPM)^4^. They are essential in improving the symbiosis between legumes and Rhizobium, which enhances nodulation and nitrogen (N_₂_) fixation^5^, as well as improves soil structure^6^. AMF also activate plant defense mechanisms or induce systemic resistance to various diseases^7,8^. Because AMF are obligate biotrophic organisms that depend on the host plant root for growth and reproduction, their large-scale production is challenging, which limits their practical application in the field^9^. Developing effective AMF-based formulations is a crucial first step toward long-term, practical use in agriculture. Microbial inoculants, particularly AMF, provide an environmental substitute for conventional fertilizers and pesticides^10^. Their efficacy is dependent on the selection of effective fungal isolates, suitable carrier materials, stimulating amendments, and application techniques that ensure microbial survival and functionality in the rhizosphere^11^.

The selection of a suitable carrier is particularly critical for AMF inoculants to maintain microbial viability and formulation stability, facilitate uniform distribution in the soil, and contribute to improved nutrient cycling and microbial diversity within the rhizosphere^12^. The carrier should maintain adequate moisture, contain a minimum amount of carbon source that ensures the survival of microbial inoculants during storage, be cost-effective and environmentally friendly, allow good adhesion to the seed, and be easy to process and sterilize^13^.

Additionally, incorporating plant biostimulants (PBs) into AMF formulations is crucial for increasing their effectiveness. The PBs are eco-friendly compounds that increase plant productivity even at low concentrations^14^. These biostimulants enhanced plant biomass production and yield when combined with AMF^15^. They also promote nutrient uptake and translocation, improving the root system, increasing soil fertility, and ultimately enhancing plant performance^16^.

In the previous study, different AMF isolates and their mixture were evaluated for their ability to form symbiosis with lupine, an economically and ecologically important legume crop valued for its high-quality protein content and contribution to sustainable agriculture^17,18^. Among the tested isolates, *E. lutea* exhibited the highest compatibility with lupine, which enhanced plant growth and resulted in significant suppression of *Rhizoctonia solani* Kühn, which is a destructive soil-borne pathogen causing damping-off and root rot and leading to considerable annual loss in crop yield^19^.

Therefore, this research was conducted to develop different formulations of *Entrophospora lutea* using peat and vermiculite as carrier materials supplemented with various stimulatory amendments and to evaluate their efficacy in controlling *Rhizoctonia solani* root rot and improving lupine growth and yield under greenhouse and field conditions. We hypothesized that incorporating selected biostimulants into *E. lutea* formulations would increase the number of infective propagules and root colonization, and consequently enhance disease suppression, plant growth, nodulation, and yield compared with the unamended *E. lutea* inoculum.

## Results

### Greenhouse experiments

In this preliminary experiment, the tested amendments differentially affected the production of infective propagules and root colonization of *E. lutea* (Table 1). Among the evaluated amendments, mannitol was the most effective, resulting in the highest production of infective propagules and root colonization. Compared with the unamended *E. lutea* inoculum, mannitol increased hyphal production by approximately 208%, spore production by 95%, and root colonization by 22%. Proline also significantly enhanced propagule production and achieved a root colonization rate comparable to that of mannitol. Humic acid, ascorbic acid, and hemicellulose increased propagule production to varying degrees; however, their effects on root colonization were less pronounced. Overall, the incorporation of biostimulant amendments improved the infective potential of *E. lutea*, with mannitol and proline showing the greatest stimulatory effects.

**Table 1:** Number of infective propagules per seed and percentage of root colonization of *Entrophospora lutea* using different amendments.

Treatments	Infective propagules per seed		Colonization rate % after 30 days
	Hypha	Spores	
AMF + (Pt + V) * + Mannitol	24.6 ± 0.84 d**	43.0 ± 0.71 e	44 ± 0.89 c
AMF + (Pt + V) + Proline	16.6 ± 1.23 c	38.6 ± 1.14 d	43 ± 1.79 c
AMF + (Pt + V) + Humic acid	16.2 ± 0.43 c	32.6 ± 1.58 c	33 ± 1.58 a
AMF + (Pt + V) + Ascorbic	16.0 ± 0.71 bc	26.6 ± 0.89 b	31 ± 1.58 a
AMF + (Pt + V) + Hemicellulose	14.6 ± 0.48 b	22.0 ± 0.71 a	30 ± 0.55 a
AMF + (Pt + V)	8.00 ± 0.84 a	22.0 ± 1.52 a	36 ± 1.58 b

*AMF + (Pt + V) = *Entrophospora lutea* (AMF) + peat (Pt) + vermiculite (V) carrier. **Values are presented as mean ± SD (standard deviation). Means in each column followed by the same letter are not significantly different according to Duncan’s multiple range test (P ≤ 0.05).

Based on the results obtained from the evaluation of individual amendments, two combined formulations were developed and assessed for infective propagule production and root colonization. Both formulations significantly enhanced *E. lutea* activity compared with the unamended inoculum, resulting in increased propagule production and root colonization. Among the tested formulations, Formulation 2 exhibited the highest numbers of hyphae and spores (26.2 and 57.2 per seed, respectively) and achieved the greatest root colonization rate (63%), followed by Formulation 1 (54%). In contrast, the unamended *E. lutea* treatment produced only 10 hyphae and 23 spores per seed, with a colonization rate of 37% (Table 2). Overall, the formulated inoculants outperformed the unamended *E. lutea* treatment, demonstrating the positive impact of amendment incorporation on inoculum performance. Furthermore, the combined application of amendments enhanced *E. lutea* activity than individual amendment treatments, indicating a synergistic effect among the incorporated amendments.

**Table 2:** Number of infective propagules per seed and percentage of root colonization of *Entrophospora lutea* formulations.

Treatments *	Infective propagules per seed		Colonization rate % after 30 days
	Hypha	Spores	
AMF + (Pt + V)	10.0 ± 0.45 a**	23.0 ± 1.14 a	37 ± 1.14 a
Formulation 1	20.6 ± 0.84 b	46.2 ± 1.14 b	54 ± 1.14 b
Formulation 2	26.2 ± 1.52 c	57.2 ± 0.84 c	63 ± 0.71 c

*AMF + (Pt + V) = *Entrophospora lutea *(AMF) + peat (Pt) + vermiculite (V) carrier. Formulation 1: *E. lutea* + peat + vermiculite amended with proline, ascorbic acid, and hemicellulose; Formulation 2: *E. lutea* + peat + vermiculite amended with proline, humic acid, and mannitol. **Values are presented as mean ± SD (standard deviation). Means in each column followed by the same letter are not significantly different according to Duncan’s multiple range test (P ≤ 0.05).

**Fig. 1:**
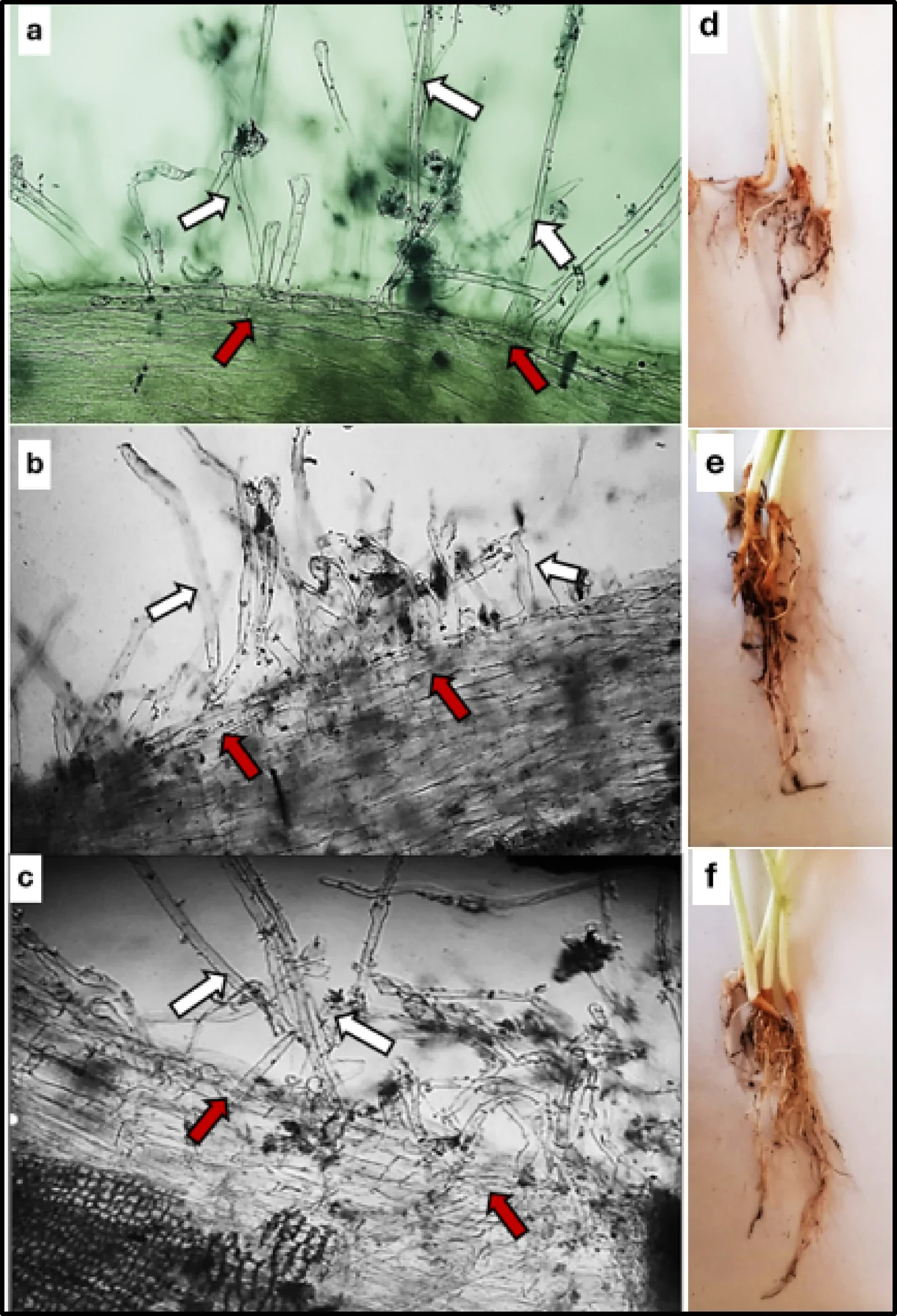
Light micrographs illustrating the colonization of *Entrophospora lutea* in lupine roots after 30 days of planting. (**a**, **d**) peat + vermiculite; (**b**, **e**) Formulation 1;(**c**, **f**) Formulation 2. White arrows point to extraradical hyphae attached to the root surface, and red arrows indicate intercellular hyphae within the root.

Microscopic observations confirmed successful colonization of *E. lutea* within the roots of lupine plants treated with the different formulations. As shown in Figures (1c, f), roots of plants treated with Formulation 2 exhibited the highest level of mycorrhizal colonization (63%, as presented in Table 2), characterized by numerous extraradical hyphae attached to the root surface and intercellular hyphae. Roots treated with Formulation 1 (Figs. 1b, e) also showed a high level of colonization (54%, as presented in Table 2), characterized by many extraradical hyphae. In contrast, roots inoculated with *E. lutea* alone (Figs. 1a, d) showed fewer extraradical hyphae.

### Impact of AMF treatments and Rizolex on the occurrence of damping-off disease of lupine plants under greenhouse conditions

Data in Table 3 indicated that all *E. lutea* treatments and the fungicide Rizolex markedly reduced pre-emergence damping-off, with only 4% dead seeds compared with 40% in the untreated infected control. A similar trend was observed for post-emergence damping-off, where Formulation 2 and Rizolex exhibited the greatest reduction (4%), followed by Formulation 1 and the unamended *E. lutea* treatment, compared with the infected control. All treatments significantly enhanced plant survival compared to infected control plants (48%), with the highest values recorded for Formulation 2 and Rizolex (92%), followed by Formulation 1 (90%) and *E. lutea* alone (88%), with 100% survival observed in the healthy control. In addition, all treatments significantly reduced disease severity compared with infected control plants (65.8%). The lowest disease index was recorded in Rizolex, closely followed by Formulation 2 and Formulation 1.

**Table 3:** Impact of different formulations of *Entrophospora lutea* and Rizolex on the occurrence of damping-off disease of lupine plants under greenhouse conditions.

Treatments*	Damping- off				Survived plants %	Increasing overinfected control %	Disease index
	Pre-emergence		Post-emergence				
	Incidence %	Reduction %	Incidence %	Reduction %			
AMF + (Pt +V)	4.0 ± 0.45 b**	90	8.0 ± 0.55 b	33.3	88 ± 0.55 c	83.3	24.7
Formulation 1	4.0 ± 0.45 b	90	6.0 ± 0.45 c	50.0	90 ± 0.55 bc	87.5	24.3
Formulation 2	4.0 ± 0.45 b	90	4.0 ± 0.45 d	66.7	92 ± 0.55b	91.7	24.2
Rizolex	4.0 ± 0.45 b	90	4.0 ± 0.45 d	66.7	92 ± 0.55 b	91.7	23.3
Control (*R. solani*)	40.0 ± 0.55a	-	12.0 ± 0.55 a	-	48 ± 0.44 d	-	65.8
Healthy Control (non-infested soil)	0.0 c		0.0 e		100 a		0.0

*AMF + (Pt +V) =* Entrophospora lutea *(AMF)+ peat (Pt) + vermiculite (V) carrier. Formulation 1: *E. lutea* + peat + vermiculite amended with proline, ascorbic acid, and hemicellulose; Formulation 2: *E. lutea* + peat + vermiculite amended with proline, humic acid, and mannitol.

**Values are presented as mean ± SD (standard deviation). Means in each column followed by the same letter are not significantly different according to Duncan’s multiple range test, (P ≤ 0.05)

### Effect of AMF treatments and Rizolex on some growth parameters of lupine plants grown under greenhouse conditions

Data in Table 4 demonstrate that all *E. lutea* treatments significantly enhanced growth parameters of lupine plants compared with the infected control. Overall, Formulation 2 produced the highest values across most growth parameters, followed by Formulation 1 compared with the unamended *E. lutea* treatment. Plant height, fresh and dry weights of shoots and roots were significantly improved by all AMF treatments, particularly Formulation 2, showing the greatest enhancement. In contrast, the infected control exhibited the lowest growth performance, while the fungicide Rizolex showed comparatively limited improvement in growth parameters. Nodulation was also significantly stimulated by AMF treatments, with the highest nodule number and nodule dry weight recorded in Formulation 2, followed by Formulation 1, compared to unamended *E. lutea* treatment. This enhancement in nodulation was associated with increased nitrogenase activity, which was highest in Formulation 2 among all treatments. Furthermore, root colonization was markedly improved in AMF treatments, with Formulation 2 achieving the highest colonization rate (78%), followed by Formulation 1 (69%). Healthy control plants grown in non-infested soil exhibited moderate values of all measured growth parameters. However, the formulated inoculants generally outperformed the unamended *E. lutea* treatment particularly with Formulation 2.

**Table 4:** Impact of different formulations of *Entrophospora lutea* and Rizolex treatments on some crop parameters of lupine plants under greenhouse conditions after 60 days of planting.

Treatments*	Plant height cm	Shoot fresh weight g/plant	Shoot dry weight g/plant	Root fresh weight g/plant	Root dry weight g/plant	Nodule number/plant	Nodules dry weight mg/plant	*N*_2_-ase activity**	Colonization rate %
AMF + (Pt + V)	40.4 ab***	15.31 b	4.40 b	12.73 b	2.43 b	41c	461.3 c	29.4	53
Formulation 1	41.5 a	14.47 c	4.38 b	12.14 c	2.34 b	44 b	490.3 b	30.9	69
Formulation 2	42.3 a	16.03 a	4.71 a	13.20 a	2.77 a	52 a	509.8 a	34.7	78
Rizolex	38.8 b	7.86 d	2.83 c	6.02 e	1.69 c	12 e	118.6 e	5.2	-
Control (*R. solani*)	23.3 d	6.17 e	1.32 e	4.18 f	1.17 d	11 e	83.5 f	4.3	-
Healthy Control (non-infested soil)	36.8 c	7.94 d	2.36 d	6.71 d	1.74 c	22 d	172.3 d	12.7	-

* AMF + (Pt +V) =* Entrophospora lutea *(AMF)+ peat (Pt) + vermiculite (V) carrier. Formulation 1: *E. lutea* + peat + vermiculite amended with proline, ascorbic acid, and hemicellulose; Formulation 2: *E. lutea* + peat + vermiculite amended with proline, humic acid, and mannitol. ** Nitrogenase activity *µ*mole C_2_H_4_/g dry nodule /h. ***Means in each column followed by the same letter are not significantly different according to Duncan’s multiple range test, (P ≤ 0.05)

**Fig. 2:**
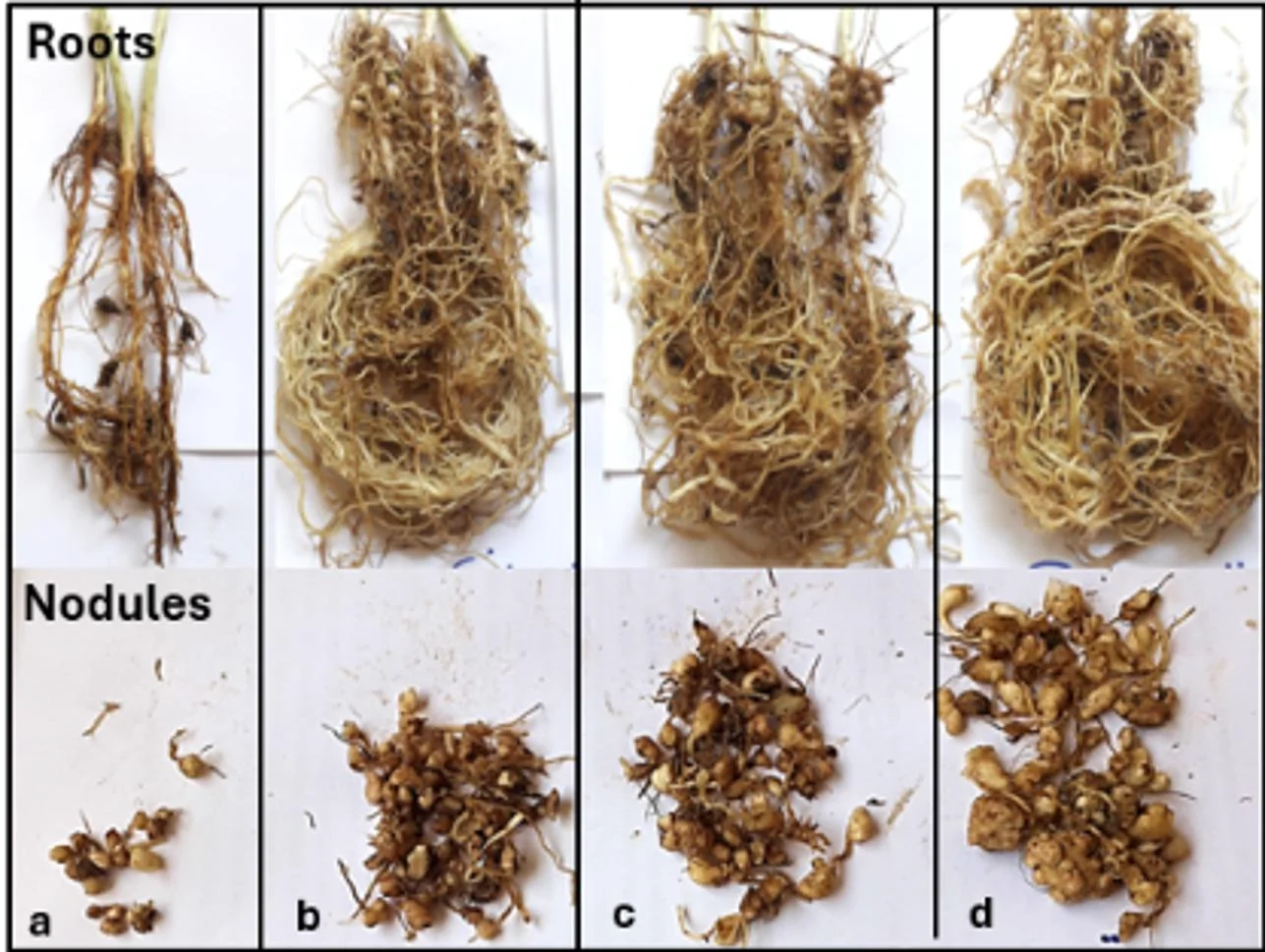
Roots and nodules of lupine plants grown in artificially infested soil with *Rhizoctonia solani*; (**a**) Control without AMF; (**b**) Lupine plants treated with *Entrophospora lutea* (peat + vermiculite); (**c**) Lupine plants treated with Formulation 1; and (**d**) Lupine plants treated with Formulation 2.

Figure 2 illustrates the influence of AMF treatment on root development and nodule formation in lupine plants grown in soil artificially infested with *R. solani*. The infected control plants without AMF (Fig. 2a) were characterized by thin and poorly developed roots with few nodules. While plants treated with *E. lutea* (peat + vermiculite) (Fig. 2b) had better developed roots with more nodules. The plants treated with Formulation 1 (Fig. 2c) showed more vigorous, branched roots with a higher number of nodules. Plants treated with Formulation 2 (Fig. 2d) showed the thickest and most extensive roots, with large and numerous nodules. These observations were consistent with the higher root colonization, nodulation, and nitrogenase activity recorded in Table 4.

Table 5 shows that all AMF-treated plants exhibited higher activities of peroxidase, polyphenol oxidase, and catalase, together with increased proline accumulation and greater H_₂_O_₂_ scavenging capacity compared with both infected and healthy controls. Formulation 2 recorded the highest values for catalase activity, proline content, and H₂O₂ inhibition. Peroxidase and polyphenol oxidase activities were also highest in Formulation 2 and the unamended *E. lutea* treatment, with no significant differences between them. Formulation 1 similarly enhanced all measured biochemical parameters compared with the controls. In contrast, the infected control exhibited the lowest antioxidant capacity, whereas the healthy control showed lower enzyme activities than AMF-treated plants. Overall, the enhanced antioxidant responses observed in mycorrhizal plants were associated with the reduced disease incidence and severity reported in Table 3.

**Table 5:** Impact of *Entrophospora lutea* treatments on the activity of oxidative enzymes, proline, and H₂O₂ inhibition % of lupine plants under greenhouse conditions.

Treatments*	Peroxidase activity Unit/mg protein/min	Polyphenol oxidase activity Unit/mg protein/min	Catalase activity Unit/mg protein/min	Proline mg/g/h/ fresh weight	H_2_O_2_ Inhibition %
AMF + (Pt + V)	0.846 a**	0.523 a	1.864 a	1.969 a	56.9 b
Formulation 1	0.812 b	0.430 b	1.922 a	2.189 a	77.5 a
Formulation 2	0.875 a	0.597 a	2.163 a	2.408 a	82.6 a
Control (*R. solani*)	0.365 c	0.204 c	0.803 c	0.795 c	10.6 d
Healthy Control (non-infested soil)	0.171 d	0.060 d	1.123 b	1.141 b	13.1 c

*AMF + (Pt +V) =* Entrophospora lutea *(AMF)+ peat (Pt) + vermiculite (V) carrier. Formulation 1: *E. lutea* + peat + vermiculite amended with proline, ascorbic acid, and hemicellulose; Formulation 2: *E. lutea* + peat + vermiculite amended with proline, humic acid, and mannitol. **Means in each column followed by the same letter are not significantly different according to Duncan’s multiple range test, (P ≤ 0.05)

## Field experiments

### Impact of AMF treatments and Rizolex on the occurrence of damping-off disease of lupine plants grown under natural conditions

Table 6 shows that all *E. lutea* treatments and Rizolex significantly reduced pre- and post-emergence damping-off and increased plant survival compared with the untreated control during both growing seasons. Among the tested treatments, Formulation 2 and Rizolex provided the greatest protection against damping-off and achieved the highest plant survival percentages in both seasons, with no significant differences between them. Formulation 1 also improved disease control and plant survival compared with the unamended *E. lutea* treatment. Overall, the incorporation of combined amendments into the inoculant improved the field biocontrol performance of *E. lutea*, particularly with Formulation 2.

**Table 6:** Impact of different formulations of *Entrophospora lutea* and Rizolex treatments on the occurrence of damping-off disease of lupine plants under natural infection.

Treatments*	Damping- off				Survived plants %	Increasing overinfected control %
	Pre-emergence		Post-emergence			
	Incidence %	Reduction %	Incidence %	Reduction %		
(A): First season 2023						
AMF + (Pt +V)	6.5 ±2.83 b**	79.2	1.3 ± 1.15 c	93.4	92.2 ±0.96 b	88.5
Formulation 1	5.0 ±0.58 b	84.0	2.5 ± 0.96 b	87.4	92.5 ± 0.50 ab	89.2
Formulation 2	5.2 ± 0.50 b	83.4	1.8 ± 0.96 c	90.9	93.0 ± 0.58 a	90.2
Rizolex	5.3 ± 0.82b	83.0	1.8 ± 0.50 c	90.9	92.9 ± 0.50 a	89.9
Control	31.3 ± 4.32 a	0.0	19.8 ± 6.45 a	0.0	48.9 ± 4.03 c	0.0
B) Second season 2024						
AMF + (Pt +V)	6.8 ± 0.96 b	78.5	1.8 ± 1.63 b	91.7	91.4 ± 0.96 b	96.6
Formulation 1	6.0 ± 0.82 bc	81.1	2.2 ± 1.50 b	89.8	91.8 ± 0.50 ab	97.4
Formulation 2	5.7 ± 1.29 c	82.0	2.1 ± 0.82 b	90.4	92.2 ± 0.58 a	98.3
Rizolex	5.7 ± 1.15 c	82.0	2.0 ± 0.96 b	90.8	92.3 ±0.50 a	98.5
Control	31.7 ± 5.51 a	0.0	21.8 ± 6.13 a	0.0	46.5 ± 1.71 c	0.0

* AMF + (Pt +V) =* Entrophospora lutea *(AMF)+ peat (Pt) + vermiculite (V) carrier. Formulation 1: *E. lutea* + peat + vermiculite amended with proline, ascorbic acid, and hemicellulose; Formulation 2: *E. lutea* + peat + vermiculite amended with proline, humic acid, and mannitol.

**Values are presented as mean ± SD (standard deviation).Means in each column followed by the same letter are not significantly different according to Duncan’s multiple range test, (P ≤ 0.05)

### Impact of AMF treatments and Rizolex on some growth parameters of lupine plants grown under natural conditions

Table 7 shows that all AMF treatments and Rizolex significantly improved growth and yield parameters of lupine plants compared with the untreated control. Similar responses were observed in 2023 and 2024 growing seasons, indicating the stability of treatment performance under natural conditions. Plant height, branch number, pods number, seed weight per plant, and 100-seed weight were all significantly enhanced by AMF inoculation. Among the tested treatments, Formulation 2 generally produced the highest values for most growth parameters and yield, followed by Formulation 1 and the unamended *E. lutea* treatment. In contrast, the untreated control exhibited the lowest performance. All AMF treatments and Rizolex also resulted in significantly higher seed yields than the untreated control in both seasons. Although Formulation 2 achieved the highest yield, no significant differences were detected among Formulation 2, Formulation 1, and Rizolex for total seed yield.

**Table 7:** Impact of different formulations of *Entrophospora lutea* and Rizolex treatments on growth parameters and yield under natural infection.

Treatments	Plant height (cm)	Number of branches/ plant	Number of pods/ plant	Seed weight/ Plant (g)	100-seed weight (g)	Seed yield (ton/fed)
(A): First season 2023						
AMF + (Pt +V) *	128.0 a**	4 b	36.5 b	42.7 b	43.1 c	2.224 a
Formulation 1	129.8 a	5 a	39.3 a	44.0 b	47.3 b	2.301 a
Formulation 2	130.8 a	5 a	40.8 a	45.9 a	49.9 a	2.346 a
Rizolex	122.0 b	3 c	34.3 c	38.1 c	36.6 d	2.214 a
Control	106.0 c	2 d	19.5 d	19.9 d	24.6 e	0.735 b
(B): Second season 2024						
AMF + (Pt +V) *	129.3 ab	4 b	37.0 ab	41.8 b	44.8 c	2.233 a
Formulation 1	131.0 a	5 a	38.9 a	43.7 a	48.6 b	2.316 a
Formulation 2	131.3 a	5 a	41.2 a	45.0 a	50.8 a	2.362 a
Rizolex	123.8 b	3 c	35.0 b	37.6 c	37.2 d	2.222 a
Control	99.0 c	2 d	18.1 c	19.6 d	22.2 e	0.703 b

*AMF + (Pt +V) =* Entrophospora lutea *(AMF)+ peat (Pt) + vermiculite (V) carrier. Formulation 1: *E. lutea* + peat + vermiculite amended with proline, ascorbic acid, and hemicellulose; Formulation 2: *E. lutea* + peat + vermiculite amended with proline, humic acid, and mannitol. **Means in each column followed by the same letter are not significantly different according to Duncan’s multiple range test, (P ≤ 0.05).

## Discussion

The application of arbuscular mycorrhizal fungi as bioinoculants represents an environmentally friendly alternative to conventional fertilizers and chemical pesticides^10^. AMF promotes plant growth, improves nutrient uptake, and increases disease resistance^20^. However, large-scale use of AMF remains limited, mainly due to difficulties related to inoculum production and storage stability^21,22^. In addition, host compatibility plays a critical role in AMF application, as some isolates are host specialists while others are generalists^23^. Typically, AMF inocula are produced in soil-based systems using host plants, which often results in variable effectiveness under field conditions. Consequently, incorporating AMF inocula consisting of spores, colonized root segments, and extraradical hyphae into an appropriate carrier can improve storage stability, handling, and application efficiency in the field^24^.

This study builds upon our previous study, in which four AMF isolates and their mixture were evaluated for controlling *Rhizoctonia* root rot in lupine and improving plant growth and yield. Among these isolates, *E. lutea* exhibited the highest efficacy and was therefore selected for further development^19^. The current study aimed to improve the performance of this promising isolate through the development of carrier-based formulations supplemented with selected biostimulatory compounds.

The results of the present study indicate that supplementing the peat-vermiculite carrier with different amendments, applied individually or in combination, increased the number of infective propagules (spores and hyphae) and improved root colonization by *E. lutea.* Moreover, the combined application of amendments enhanced the efficacy of the formulation more than individual amendments alone, resulting in a significantly higher rate of root colonization by *E. lutea*. Accordingly, root colonization is considered a key indicator of AMF effectiveness and symbiotic compatibility in legumes^25,26^.

Furthermore, both formulations developed in this study enhanced the activity of *E. lutea* against *R. solani*, significantly reduced damping-off incidence and root rot severity and improving plant survival under greenhouse and field conditions compared with the unamended inoculum. In addition, they promoted lupine growth and increased seed yield. However, the second formulation, amended with proline, humic acid, and mannitol, consistently outperformed the first formulation amended with proline, ascorbic acid, and hemicellulose. Since the efficacy of *E. lutea* in suppressing *R. solani* and promoting lupine growth has already been comprehensively demonstrated and discussed in our previous study^19^, the present discussion focuses mainly on the contribution of the incorporated additives to the improved performance of the formulated inoculants.

The enhanced performance of the formulated inoculants is likely attributable to the combined action of the incorporated amendments rather than to any individual component alone. These additives have acted synergistically to produce the highest numbers of hyphae and spores and the highest root colonization rate, resulting in greater disease suppression and plant growth promotion than the unamended inoculum.

To better understand the factors underlying this enhanced performance, it is important to consider not only the effects of the incorporated amendments but also the contribution of the carrier, which plays a fundamental role in facilitating AMF establishment and ensuring effective delivery of propagules to the host plant. The peat–vermiculite carrier used in this study contributed to the successful establishment of *E. lutea* without adversely affecting lupine seed germination. This result aligns with previous reports demonstrating the effectiveness of peat-vermiculite mixtures in microbial formulation systems that promote plant growth and enhance disease tolerance^27^, as well as their strong compatibility with AMF propagules^28^. Peat remains a suitable carrier because of its high porosity, rich organic carbon content that supports microbial survival, and good compatibility with AMF propagules^29,30^. Likewise, vermiculite is an excellent inorganic carrier due to its neutral pH, low toxicity, and limited contamination risk, with superior aeration and moisture retention. It also supports microbial survival and early root development^31^. In addition, the incorporation of carboxymethyl cellulose (CMC) improved inoculum adhesion and stability during seed application, thereby enhancing inoculant retention and overall formulation quality^28,32^.

Among the incorporated amendments, mannitol contributed to the greatest increase in hyphal growth, spore production, and root colonization, particularly when applied at low concentrations in combination with other amendments, suggesting a synergistic effect on AMF establishment. This effect may be attributed to the ability of mannitol to stimulate AMF growth by promoting hyphal extension and colonization^33^. Similarly, Kuwada et al.^34^ reported that low concentrations of mannitol enhance hyphal extension and root colonization, thereby improving the establishment and performance of arbuscular mycorrhizal symbioses, whereas higher concentrations are less effective.

In the current study, proline supplementation also resulted in a high number of hyphae and spores and enhanced root colonization, reaching 44%. Furthermore, the addition of low concentrations of proline to the two tested formulations enhanced the activity of *E. lutea* under greenhouse and field conditions, likely due to its role in maintaining cellular redox balance and stabilizing cellular structures under stress conditions^35^. However, excessive proline levels can inhibit pyrroline-5-carboxylate synthase, the key enzyme involved in proline biosynthesis, resulting in reduced plant growth^36,37^. Accordingly, proline concentrations above 40 mM may limit plant growth, whereas lower concentrations (2.5–5 mM) promote growth^38,39^. These findings are consistent with our results, confirming that low proline concentrations can enhance AMF performance without adverse physiological effects.

In this study, the use of low concentrations of humic acid also improved AMF performance and root colonization. These findings are consistent with previous studies showing that low concentrations of humic acid enhance the performance of AMF inoculants, whereas higher concentrations may adversely affect AMF performance^40^. In addition, low concentrations have been reported to increase biomass accumulation and nutrient uptake^41,42^. Humic acid also stimulates AMF growth and reproduction by promoting spore formation, extraradical mycelium development, and root colonization^41,43,44^. Furthermore, it can serve as a carbon source that supports microbial survival in the soil^45^. In addition to its effects on AMF, humic acid directly promotes plant growth by improving nutrient uptake^46^ and indirectly by forming stable complexes with metallic cations, such as Zn, Mn, Cu, and Fe, thereby maintaining their availability for plant uptake^47^.

Additionally, ascorbic acid showed moderate stimulatory effects compared with the other additives, even when combined with proline and hemicellulose. Although ascorbic acid is a well-known antioxidant that enhances plant stress tolerance and scavenges reactive oxygen species (ROS)^48,49^, its interaction with proline metabolism may explain its relatively weaker performance. Ascorbic acid acts as a cofactor of proline hydroxylase, the enzyme that converts proline into hydroxyproline, which reduces proline accumulation and disrupts cellular redox balance and osmo-protective metabolism^50,51^.

Likewise, our results indicated that hemicellulose exhibited a moderate positive effect on root colonization and propagule production under greenhouse conditions, either alone or in combination with other amendments. Under field conditions, however, its positive effects became apparent over time, particularly in the combined treatment, which ultimately performed better than the unamended isolate. Although organic matter generally promotes AMF development and hyphal growth^52,53^, its effect depends on the degree of decomposition. Cellulose-rich materials decompose slowly and may temporarily suppress AMF activity^54,55^.

In the present study, hemicellulose slightly reduced plant fresh and dry weights under greenhouse conditions but improved growth parameters and yield under field conditions, indicating a time-dependent effect. As hemicellulose gradually decomposes, it may create a more favorable environment for AMF development through interactions with saprophytic microorganisms. Similarly, Gryndler et al.^56^ reported an initial decrease in AMF spore numbers following cellulose addition, followed by a significant increase after prolonged incubation. These findings suggest that cellulose-rich amendments may initially suppress AMF activity but subsequently enhance sporulation and root colonization as decomposition progresses.

The better performance of Formulation 2 compared with Formulation 1 and the unamended inoculum suggests that its efficacy was not attributed to the effect of a single additive. Although each amendment may individually enhance AMF performance, the greater efficacy observed in Formulation 2 indicates that the combined interactions among the incorporated amendments have generated synergistic effects that enhanced propagule production, root colonization, plant growth, and disease suppression than those obtained with the individual amendments alone. These findings are consistent with previous studies demonstrating that the combined application of bio-stimulants often results in synergistic effects. For example, combinations such as seaweed extracts rich in mannitol with humic acid significantly enhance shoot and root growth, photosynthetic efficiency, and yield in cereals^57–59^. Similarly, the combined application of proline and humic acid has enhanced yield components and nutrient uptake while reducing toxic element accumulation in several crops^60,61^. In addition, combined applications of proline and ascorbic acid have been reported to improve stress tolerance and productivity under saline conditions in wheat and tomato^62,63^. Overall, the findings suggest that synergistic interactions among biostimulants contributed to the enhanced performance of the formulated *E. lutea* inoculants in the present study.

Taken together, the present findings suggest that enhanced root colonization not only improved the establishment of the mycorrhizal symbiosis but also stimulated antioxidant defense mechanisms. The present study demonstrated that all AMF treatments significantly enhanced the activities of antioxidant enzymes, including peroxidase, polyphenol oxidase, and catalase, along with increased proline accumulation and reduced H₂O₂ levels, particularly with Formulation 2. These coordinated responses suggest a strengthened antioxidant defense system associated with improved AMF colonization^64^. Rather than acting independently, antioxidant enzymes function as an integrated defense network that mitigates oxidative stress through reactive oxygen species (ROS) detoxification and minimizing oxidative damage to plant tissues^65^. In this context, AMF may activate local and systemic defense responses, including systemic acquired resistance (SAR), leading to the stimulation of key enzymes such as peroxidase and polyphenol oxidase^1,65^. In line with this, polyphenol oxidase (PPO) oxidizes ortho-diphenols into o-quinones and strengthens cell walls during pathogen attack^66^, while peroxidases (PO) contribute to phytoalexin synthesis and produce antifungal ROS that limit disease development^67^. Additionally, mycorrhizal inoculation significantly enhances catalase activity and promotes the antioxidant system by accelerating ROS scavenging, which increases stress tolerance and activates plant defense responses^68^. Catalase is a key component of the ROS-scavenging by rapidly converting H₂O₂ into water and oxygen^69^. Similar responses have been reported in AMF-inoculated plants, where enhanced antioxidant enzyme activity and proline accumulation were associated with improved stress tolerance and growth^70^.

Importantly, the improved root colonization observed under greenhouse conditions was accompanied by enhanced nodulation and nitrogenase activity, reduced disease severity, and increased plant growth and yield supporting a functional synergy between AMF and rhizobia. This symbiotic interaction enhances nitrogen acquisition and nodule development, thereby improving plant nutritional status and contributing to enhanced growth and productivity in legumes^71^. These findings suggest that root colonization served as a central component linking the physiological and protective responses observed in lupine plants.

## Conclusion

In conclusion, the present study demonstrated that the formulation of *E. lutea* with selected bio-stimulatory amendments significantly enhances its performance compared with the unamended inoculum. The formulated inoculants improved propagule production, root colonization, nodulation, antioxidant defense responses, disease suppression, and yield in lupine under greenhouse and field conditions. Among the tested treatments, Formulation 2 consistently showed the most pronounced effects, highlighting the importance of amendment combinations in improving AMF performance. These findings suggest that the observed benefits are not attributable to individual components alone, but rather to the synergistic interactions among the incorporated amendments particularly mannitol, proline and humic acid which collectively enhanced symbiotic establishment and plant physiological responses.

However, certain limitations should be acknowledged. The present study was conducted under greenhouse and field conditions over two seasons; however, longer-term studies are still required to assess the stability of the formulated inoculants under diverse environmental conditions, the shelf-life and storage stability of the formulations, which is critical for large-scale production and commercial application.

## Methods

### Lupine cultivar

Lupine seeds cv. Giza 2 were obtained from the legume Research Department, Field Crops Institute, Agricultural Research Center (ARC), Giza, Egypt.

### Pathogen

The fungus *Rhizoctonia solani* with accession number PV476529.1 was obtained from naturally infected lupine plants showing typical damping-off and root rot symptoms collected from Sakha, Kafr El-Sheikh Governorate^19^. This isolate was maintained on malt extract agar slants under phosphate buffer (pH 6.5) and stored at 4 ± 0.5 °C^72^. Inoculum of *R. solani* was prepared according to the method described by Atwa et al.^73^. The fungus was grown in 500-cc glass bottles containing sterilized sorghum medium (100 g sorghum grains and 90 mL water). The bottles were inoculated with equal-sized agar discs (0.5 cm diameter) taken from 4-day-old cultures of *R. solani* and incubated at 24 ± 1 °C for 21 days. During the incubation period, the bottles were shaken for 3 min every three days to ensure uniform distribution of fungal growth. After incubation, the colonized sorghum medium was air-dried for 3 days and then ground in a mill to pass through a 3-mm sieve. The resulting inoculum was incorporated into the soil within one week of preparation.

## Root-nodule bacteria treatment

A *Rhizobium* formulation (*Bradyrhizobium* sp. *Lupinus*) was obtained from the Biofertilizers Production Unit, Soils, Water and Environment Research Institute (SWERI). The inoculant was applied at a rate of 5 g per pot at the time of sowing. For field experiments, approximately 50 kg of moistened fine sandy soil was thoroughly mixed with 800 g of the rhizobial formulation per feddan and placed into the seed furrow during sowing^19^.

### Fungicidal treatment

Lupine seeds were treated with Rizolex 50% WP (Tolclophos-methyl), supplied by Sumitomo Chemical Company Ltd., at the recommended rate of 3 g/kg of seed, using a 1% methylcellulose solution as an adhesive.

### Greenhouse experiments

The greenhouse experiment was conducted during November and December at the greenhouse of the Plant Pathology Research Institute, ARC, Giza, Egypt. During this period, the average temperature in Giza ranged from 21 to 22 °C, and the average relative humidity was approximately 61–66%.

### Seed preparation

Lupine seeds were disinfected with 1% sodium-hypochlorite solution for 2 min, finally washed with sterile distilled water, and air dried.

### AMF isolate and inoculum preparation

The AMF isolate *Entrophospora lutea* (formerly *Glomus luteum*) with accession number PP069247^19^ was used in the current study. The AMF spores were collected and isolated using the wet sieving technique^74^. The fungus was propagated for three months in multispore pot cultures containing a 3:1 (w/w) mixture of autoclaved Holland peat moss and vermiculite, with Sudan grass serving as the host plant. After harvesting, the inoculum was sieved through a 500-µm mesh to remove debris. Lupine seeds were treated with AMF inoculum using a 1% carboxy methylcellulose solution as an adhesive^75^.

### Assessment of infective propagules

Greenhouse experiments were conducted to assess the impact of various amendments on the performance of the AMF isolate *E. lutea*. To evaluate each amendment individually, each additive was tested in a separate experiment. Each experiment consisted of a fresh 10-kg batch of the peat (Pt) + vermiculite (V) carrier (3:1, w/w). The batches were amended individually with mannitol (0.5 g)^34^, proline (1.5 g)^39^, humic acid (1.5 g)^42^, hemicellulose (5 g)^56^, and ascorbic acid (0.5 g).

Based on the results obtained from the individual amendments experiments, two complete formulations were developed for further evaluation. Initially, their effect on the performance of the *E. lutea* isolate:

- Formulation ①: (Pt + V) + proline + ascorbic acid + hemicellulose.
- Formulation ②: (Pt + V) + proline + humic acid + mannitol.

After the inoculum was produced, it was mixed with 1% methylcellulose, which served as a coating material for the seeds.

To quantify the number of infective propagules, five replicates were used for each treatment. For each replicate, five lupine seeds coated with the amended inoculum were randomly collected and gently washed to detach spores and hyphal fragments. The wash water was passed through a 38-µm mesh sieve (No. 400) to retain infective propagules. The retained material was transferred to Petri dishes, and the numbers of spores and hyphae were counted separately using a stereomicroscope at 40× magnification. Infective propagule counts were recorded for each amendment and for both complete formulations^22^.

### Pot preparation and cultivation

Thirty-centimeter diameter pots were sterilized using a 5% formalin solution and left for two weeks until complete formalin evaporation. Pots were filled with steam-disinfected sandy clay soil (1:2 v/v) following the procedure described by Atwa^76^.

Soil infestation was done by mixing the inoculum of *R. solani* at the rate of 2% of soil weight^77^. The infested soil was mixed thoroughly and watered every 2 days for a week before planting to stimulate the fungal growth and ensure its distribution in the soil. Five seeds of treated lupine seeds were sown in each pot, and the pots were arranged in a completely randomized design (CRD), with twelve pots assigned to each treatment as follows:

1. *Entrophospora lutea* + peat (Pt) + vermiculite (V) carrier in infested soil.
2. Formulation 1: *E. lutea* + (Pt) + (V) carrier amended with proline, ascorbic acid, and hemicellulose in infested soil.
3. Formulation 2: *E. lutea* + (Pt) + (V) carrier amended with proline, humic acid, and mannitol in infested soil.
4. Fungicide: Rizolex in infested soil.
5. Seeds coated with (Pt) + (V) in infested soil (untreated control).
6. Seeds coated with (Pt) + (V) in non-infested soil (healthy control).

### Plant growth assessment

Sixty days after planting, twelve plants (four replicates per treatment) were carefully uprooted. Roots were gently washed under water to remove soil particles and debris. Nodules were detached from both main and lateral roots, and the number of nodules per root system was recorded. Shoot length was measured after cutting shoots at the soil surface. Shoots, roots, and nodules were then placed in paper bags and oven-dried at 70 °C for 48 h, then weighed, and the averages were recorded. Nitrogenase activity was assessed using the acetylene reduction assay according to the method described by Hardy et al.^78^. Gas samples were analyzed using a 5890 Series II Plus gas chromatograph (Hewlett-Packard, USA).

### Mycorrhizae colonization of lupine root

Root colonization by the mycorrhizal isolate was evaluated at 30 and 60 days after sowing. Roots were stained following the method described by Koske, & Gemma^79^. For each sample, five groups of ten root segments (1 cm each) were randomly selected and examined under a light microscope for the presence of AM fungal structures. Root colonization percentage was calculated according to the following formula provided by Phillips & Hayman^74^:

$$Root\;colonization\% = \frac{{\:No.\:of\:colonized\:root\:fragments\:}}{{No.\:of\:total\:root\:fragments}} \times 100$$

### Enzyme activity assay

Fifteen days after sowing, fresh plant tissue (4 g) was homogenized at 0 °C in 6 mL of 0.1 M phosphate buffer (pH 7.0) using a small amount of neutral sand to aid grinding. The homogenate was then filtered and centrifuged using a refrigerated centrifuge (Sigma 4K15, Sigma Laborzentrifugen GmbH, Germany) at 3000 rpm for 15 min at 4 °C. The resulting supernatant, representing the crude enzyme extract, was either used immediately for enzyme analysis or stored at − 20 °C until further use.

### Assay of peroxidase (PO)

PO activity was measured according to Chakraborty and Chatterjee^80^. The reaction mixture contained 1.5 mL of enzyme extract and 5 mL of freshly prepared pyrogallol reagent. The absorbance was adjusted to zero before initiating the reaction with 0.5 ml of 1% H_2_O_2_. Peroxidase activity was determined spectrophotometrically by recording the increase in absorbance at 430 nm per minute.

### Assay of polyphenol oxidase (PPO)

PPO activity was assessed following the method described by Sadasivam and Manickam^81^. For the assay, 2 mL of the enzyme extract was combined with 3 mL of phosphate buffer in a cuvette, and the spectrophotometer was set to zero at 495 nm. The reaction was then initiated by adding 1 mL of 0.01 M catechol prepared in phosphate buffer (0.4 mg/mL). PPO activity was expressed as the rate of increase in absorbance per minute at 495 nm.

### Assay of catalase (CAT)

CAT activity was determined according to Aebi^82^. The reaction mixture contained 1.5 mL of 50 mM potassium phosphate buffer (pH 7.0), 0.1 mL of enzyme extract, and 0.2 mL of freshly prepared 100 mM hydrogen peroxide (H_2_O_2_). The reaction was initiated by adding H_2_O_2_. CAT activity was expressed as the change in absorbance per minute at 240 nm.

### Assay of hydrogen peroxide (H_2_O_2_) scavenging

H_2_O_2_ scavenging activity was determined according to Manju and Pushpa^83^ with slight modifications. A solution of H₂O₂ (40 mM) was prepared in phosphate buffer (pH 7.4). Leaf extract was prepared by dissolving 1 mg of the methanolic extract in 10 mL of methanol, and 1 mL of this mixture was mixed with 0.6 mL of the H_2_O_2_ solution. The final reaction volume was adjusted to 3 mL with phosphate buffer. The final mixture was incubated at room temperature for 10 min, and the absorbance was recorded at 230 nm. A blank (A₀) was prepared following Green and Hill^84^ using phosphate buffer without H_2_O_2_. The absorbance of the reaction mixture containing extract and H_2_O_2_ was recorded as A₁. The percentage of H_2_O_2_ scavenging activity was calculated using the formula:

$$\:{\mathrm{H}}_{2}{\mathrm{O}}_{2}\mathrm{\:scavenging\:activity\:(\%)}=\frac{{A}_{0}-{A}_{1}}{{A}_{0}}\times\:100$$

### Determination of proline content

Proline content was determined according to Bates et al.^85^. Approximately 0.5 g of fresh leaves (40 days after sowing) were ground in liquid nitrogen and extracted in 3% sulfosalicylic acid. The homogenate was centrifuged at 8000 rpm for 10 min at 4 °C, and 2 mL of the resulting supernatant were mixed with 2 mL of the reagent consisting of a 1:1 (v/v) mixture of ninhydrin and glacial acetic acid. The mixture was incubated at 100 °C for 40 min, and the reaction was terminated in an ice bath. The reaction mixture was extracted with 4 mL of toluene, and the absorbance was measured at 520 nm.

### Disease incidence and disease severity

The disease incidence (DI) of pre- and post-emergence damping-off was recorded 15 and 30 days after planting. The survival plants were recorded after 45 days under greenhouse and field conditions^76^.

The percentage of reduction or increase relative to the infected control was calculated as follows:

$$\operatorname{Reduction}{{ }}\:or{{ }}\:increasing\:{{ }}\% \: = \:\:\:\frac{{\:\left( {DI} \right)\:of\:\:infected\:control\: - \:DI\:of\:treatment\:\:}}{{DI\:of\:\:infected\:control}}\:x100\:$$

A numerical rating scale (0–5) was used to rate the plants for disease severity^86^, and the following formula was used to determine the disease index of root rot:

$$Disease\:{{ }}Index\: = \:\:\frac{{\sum {\left( {number\:of\:roots\:tested\:in\:each\:grade\:x\:\:\deg ree\:of\:damage\:[0 - 5]} \right)} }}{{total\:number\:of\:tested\:plants\:x\:the\:highest\:\deg ree\:of\:\:infection\:\left( 5 \right)}} \times 100$$

### Field experiments

The field trials were carried out at Giza Agricultural Research Station, ARC, Egypt. A randomized block design (RBD) with five treatments and four replicates per treatment (20 plots) was used for the experiment during the 2023 and 2024 seasons at the beginning of November. The area of each plot was 10.5 m² and consisted of five rows. Each row was 3.5 m in length and 0.6 m in width. Two lupine seeds were sown at a row spacing of 20 cm apart on either side of the row ridge. The treatments were as follows:

1. *Entrophospora lutea* + peat (Pt) + vermiculite(V) carrier.
2. Formulation 1: *E. lutea* + (Pt) + (V) carrier amended with proline, ascorbic acid, and hemicellulose.
3. Formulation 2: *E. lutea* + (Pt) + (V) carrier amended with proline, humic acid, and mannitol.
4. Fungicide: Rizolex.
5. Untreated control: Seeds coated with (Pt) + (V).

All agricultural practices, including irrigation and fertilization, were carried out according to the recommendations of the Egyptian Ministry of Agriculture. At harvest, ten lupine plants were randomly selected from the inner rows of each plot to record growth characteristics and estimate seed yield (tons per feddan).

### Statistical analysis

Data were analyzed using ASSISTAT statistical software developed by Silva and Azevedo^87^. Analysis of variance (ANOVA) was performed, and treatment means were compared using Duncan’s multiple range test at a significance level of *P* ≤ 0.05. Means in each column in the tables followed by the same letter are not significantly different.

## Data Availability

All data generated or analyzed during this study are included in this published article.
